# Analysis of 1840 Equine Intraocular Fluid Samples for the Presence of Anti-*Leptospira* Antibodies and Leptospiral DNA and the Correlation to Ophthalmologic Findings in Terms of Equine Recurrent Uveitis (ERU)—A Retrospective Study

**DOI:** 10.3390/vetsci9080448

**Published:** 2022-08-21

**Authors:** Tobias Geiger, Hartmut Gerhards, Bogdan Bjelica, Elke Mackenthun, Bettina Wollanke

**Affiliations:** 1 Clinic for Horses, University of Veterinary Medicine, 30559 Hanover, Germany; 2 Equine Clinic, Clinical Department, Ludwig-Maximilians-University (LMU), 85764 Oberschleissheim, Germany; 3 Department of Neurology, Hanover Medical School, 30625 Hanover, Germany; 4 Equine Clinic at the Racetrack, An der Rennbahn 16, 76473 Iffezheim, Germany

**Keywords:** equine recurrent uveitis (ERU), *Leptospira* spp., intraocular infection, microscopic agglutination test, ELISA, LipL32, PCR, aqueous and vitreous samples, vitrectomy

## Abstract

**Simple Summary:**

In horses, the chronic intraocular leptospiral infection has been shown to cause equine recurrent uveitis (ERU). This inflammatory ophthalmic disease recurs for years and usually leads to blindness. Only recently it was found that biofilm formation of the leptospires in the vitreous cavity leads to uveitis recurrences and prevents effective elimination of the infection by antibiotics or by the immune system. The most effective treatment is vitrectomy (lavage of the vitreous cavity), which mechanically removes the biofilm infection. This surgery has been performed in horses for more than 30 years, and thousands of intraocular specimens have been analyzed for antibodies directed against leptospires and by PCR for leptospiral DNA. For the present study, medical records were retrospectively analyzed. Complete medical and laboratory records were available for 1800 intraocular specimens from horses treated from 2002 to 2017 (1387 specimens from ERU-eyes, 237 specimens from eyes affected with another type of uveitis, and 216 specimens from healthy eyes). In 83% of intraocular samples from ERU eyes, antibodies were detectable, and especially the detection of immunoglobulin A (IgA) seems to play an important role. In 72% of the intraocular specimens, leptospiral DNA was detectable by PCR. No antibodies were detectable in the samples from eyes with another type of uveitis or in the samples from healthy eyes. A PCR was positive in only one sample from a healthy eye. These results with a very high number of intraocular specimens demonstrate the great importance of an intraocular leptospiral infection for ERU. It can be concluded that for a reliable diagnosis of intraocular leptospiral infection or to reliably exclude an infection, multiple tests should be applied.

**Abstract:**

In the equine clinic of the LMU in Munich, therapeutic vitrectomies have been routinely performed in horses for three decades. The vitreous samples obtained during vitrectomies were usually tested for anti-*Leptospira* antibodies and for more than 20 years also by PCR for leptospiral DNA. If the indication for surgery was ophthalmologically inconclusive, an aqueous humor was collected preoperatively and examined for evidence of leptospiral infection. In this study, medical records from 2002 to 2017 were analyzed. Records for 1387 eyes affected by equine recurrent uveitis (ERU) and 237 eyes affected by another type of uveitis met the inclusion criteria. A total of 216 samples from healthy eyes were used as controls. In 83% of intraocular samples from ERU eyes, antibody titers of 1:100 or higher were detectable by microscopic agglutination test (MAT). Similarly, 83% of intraocular samples had anti-*Leptospira* antibodies detected by ELISA. In 72% of the intraocular specimens, leptospiral DNA was detectable by PCR. No antibodies were detectable in the samples from eyes with another type of uveitis or in the samples from healthy eyes. A PCR was positive in only one sample from a healthy eye. These results with a very high number of intraocular specimens demonstrate the great importance of an intraocular leptospiral infection for ERU. It can be concluded that for a reliable diagnosis of intraocular leptospiral infection or to reliably exclude an infection multiple tests should be applied.

## 1. Introduction

As in other species, various types of uveitis occur in horses. The preliminary report and a careful clinical and ophthalmologic examination allow an etiologic diagnosis of uveitis in most cases [1]. In addition to traumatic uveitis, which typically occurs only once [2,3], there is also chronic insidious uveitis (e.g., uveitis in leopard coat pattern horses [3,4,5], phacogenic uveitis [6], chronic iritis [1,7] or tumor-associated uveitis [8,9,10], therapy-resistant uveitis (e.g., in intraocular parasitosis [11,12]) or septicemia-associated uveitis (most frequently in foals with rhodococcosis [13]). Regardless of etiology, each acute uveitis episode requires well-aimed and meticulous conservative therapy [3,10]. Whenever possible, the underlying disease must be treated (e.g., septicemia in foals). 

In chronic uveitis, various therapeutic options have been described to prevent progressive damage to intraocular structures and further relapses (e.g., subscleral cyclosporine implants [14] or intravitreal gentamicin injections [15,16,17,18,19]), which, however, should be applied selectively and depending on the etiology of the uveitis [1]. 

Typical equine recurrent uveitis (ERU) is characterized by painful recurrent episodes of uveitis at unpredictable intervals [3,20] and is caused by a chronic intraocular leptospiral biofilm infection [1,21]. Most equine eyes affected with ERU will lose vision over time unless further episodes of uveitis can be prevented [3,22]. In about one-third of horses, both eyes are affected [1,23]. The incidence of ERU in Europe is reported to be 7–10% [24,25]. Thus, ERU plays a challenging role in equine medicine and represents a great economic burden.

Studies from the USA showed an even greater incidence of up to 25% [3]. This high incidence in the U.S. can be partially explained by the inconsistent use of the term “ERU” in the literature. Often horses with glaucoma and horses with the insidious uveitis typical of leopard coat pattern horses are referred to as “ERU” [3,26]. In these diseases, however, the recurrent episodes of uveitis typical of ERU do not occur. In the following, the term “ERU” will therefore be used exclusively for the type of uveitis in horses that is associated with the typical recurrent and painful episodes of uveitis as well as the typical ophthalmological changes [1]. 

The most effective treatment option for ERU is vitrectomy (irrigation of the vitreous cavity) [27], which removes the leptospiral biofilm from the eye [1]. This operation not only leads to the permanent absence of recurrence in up to 97% of the operated eyes but can also serve to permanently preserve vision in the affected equine eyes if performed early enough in the course of the disease [28,29,30,31]. In addition, vitrectomy allows examination of the removed vitreous material [20,32,33,34,35,36,37].

Of course, vitrectomy is also associated with risks that can lead to blindness [36]. However, when surgery is properly performed by an experienced equine ophthalmic surgeon and when equipment (devices and instruments) suitable for equine eyes is used, serious complications are very rare [28]. In any case, the risk of blindness is incomparably higher without vitrectomy, and a properly conducted vitrectomy shortens the suffering from a very painful eye disease.

The present retrospective study of a very large number of equine eyes affected with ERU and the laboratory results of specimens from these eyes intends to provide data illustrating the importance of intraocular leptospiral infection in ERU and to demonstrate the laboratory diagnostic possibilities with intraocular specimens to determine the best possible therapy in each case of equine uveitis.

## 2. Materials and Methods

### 2.1. Search of Medical Records

Medical records of the Equine Clinic of the Ludwig Maximilian University (LMU) from 2002 to 2017 were searched for horses from which aqueous humor or vitreous samples had been tested for anti-*Leptospira* antibodies and leptospiral DNA. For this study, all samples from eyes affected by keratitis or glaucoma were excluded. In addition, only those medical records were used in which the medical records and documentation were complete (preliminary report, documentation of ophthalmologic findings obtained at the clinic, and laboratory results). 

Finally, of well over 2500 medical records in which the examination of intraocular specimens had been documented, only 1624 met the inclusion criteria for this study. Of these intraocular samples, 1387 originated from eyes affected by ERU and 237 from eyes with another type of uveitis (e.g., phacogenic uveitis, uveitis in leopard coat pattern horses, traumatic uveitis, or chronic iritis). 

Diagnosis of ERU or ruling out ERU required a complete ocular examination by a senior equine clinician well-experienced in ophthalmology and/or the examination of an intraocular sample (either vitreous or aqueous humor). The clinical diagnosis of “ERU” was made on the basis of ophthalmologic findings, as described previously [1,38]. 

If the medical history and the ophthalmologic findings were conclusive, vitrectomy was performed without prior examination of an intraocular sample. If ERU was suspected but the eyes did not yet show clear evidence of ERU on ophthalmologic examination, aqueous humor was collected first. The aqueous humor was then sent to an external laboratory and analyzed for anti-*Leptospira* antibodies by a microscopic agglutination test (MAT) and often additionally by an in-house ELISA. For budgetary reasons, ELISA was often only performed as a supplementary test if MAT was negative. In addition, PCR (targeting LipL32 or 16 s RNA) was also performed in many cases. If any of these laboratory tests yielded a positive result, the diagnosis of ERU was considered confirmed. 

The control group comprised 216 intraocular samples from healthy eyes from horses that had to be euthanized for reasons other than eye disease (e.g., because of colic, injuries, or orthopedic conditions). The ophthalmological examination was performed either before or immediately after euthanasia. These samples from healthy eyes had been routinely collected at times to have controls for earlier investigations [4,39,40]. 

### 2.2. Equine Patients

The age of horses with ERU ranged from 9 months to 23 years (mean 12 years). Control horses ranged in age from 1 to 25 years (mean 13 years). A total of 380 mares (control group: 59 mares), 471 geldings (control group: 73 geldings), and 94 stallions (control group: 12 stallions) of different colors and breeds were represented. The horses mainly came from Germany, although several came from neighboring countries such as Austria, Switzerland, Poland, the Czech Republic, France, Belgium, the United Kingdom, and the Netherlands.

The total duration of the disease, the number of uveitic attacks observed, and the period of time that had passed since the last inflammatory episode was extracted from the medical history. In the 216 healthy control eyes, previous ocular disease or irritation was excluded as far as possible by anamnesis and ophthalmological examination. 

### 2.3. Intraocular Samples

Of the 1840 intraocular samples, laboratory results were used from either the aqueous humor or vitreous samples, never two from one eye. 

In this study, 1433 vitreous samples were analyzed. Thereof, 1168 vitreous samples were obtained during therapeutic vitrectomies. All vitrectomies were performed as described by Gerhards and coworkers [27]. A three-way stopcock attached to the suction line allowed the sterile withdrawal of 3–4 mL of undiluted vitreous material with a 5 mL syringe at the beginning of surgery and before the infusion line was opened for the lavage fluid. Vitreous samples from healthy control eyes were collected immediately after euthanasia using a sterile intravenous catheter.

Furthermore, 407 aqueous specimens were analyzed. Thereof, 32 belonged to healthy eyes from horses in the control group. The other 375 aqueous humor samples were collected before vitrectomy to determine whether evidence of intraocular leptospiral infection was present and thus whether surgery was indicated. In these horses, 0.5–1.0 mL of aqueous humor was extracted by limbal paracentesis of the anterior chamber with a 2 mL syringe and a 27 G hypodermic needle under short general anesthesia. In the healthy control horses, the aqueous samples were obtained in the same way immediately after euthanasia.

### 2.4. Laboratory Analysis

The MAT was performed in accordance with the guidelines of the O.I.E. (Office International des Epizooties, The World Organisation for Animal Health, OIE 2014). The following leptospiral serovars (WHO-strains) were used as live antigens: *L. interrogans* serogroup Australis serovar Bratislava, *L. interrogans* serogroup Canicola serovar Canicola, *L. interrogans* serogroup Icterohaemorrhagiae serovar Copenhageni, *L. interrogans* serogroup Grippotyphosa serovar Grippotyphosa, *L. interrogans* serogroup Sejroe serovar Hardjo, *L. interrogans* serogroup Icterohaemorrhagiae serovar Icterohaemorrhagiae, *L. interrogans* serogroup Javanica serovar Javanica, *L. interrogans* serogroup Pomona serovar Pomona, *L. interrogans* serogroup Pyrogenes serovar Pyrogenes, *L. interrogans* serogroup Sejroe serovar Saxkoebing, *L. interrogans* serogroup Sejroe serovar Sejroe, and *L. interrogans* serogroup Tarassovi serovar Tarassovi. The cut-off titer for a positive MAT result was considered 1:100 or higher. Often, co-reactions of different serovars were present in the MAT. However, only the serovar with the highest MAT titer was included in the analysis. To facilitate statistical analysis, the respective leptospiral serovar was not considered further.

The serovar-specific in-house ELISA technique was developed by Kettner [41] and was applied to 892 intraocular specimens. For this in-house ELISA test, an antigen extraction from the respective pathogenic serovars must be performed first. This indirect technique, using a complete leptospiral antigen, allowed both qualitative and quantitative determination of the immune response because the individual immunoglobulin classes, i.e., IgM, IgG, and IgA, were detected separately with anti-horse IgA, anti-horse IgG, and anti-horse IgM, and could reach different values. Thus, a sample could yield a negative (−), borderline (+/−), weakly positive (+), positive (++), or strongly positive (+++) result, depending on the optical density determined by photometry (wavelength 405 nm). In further analysis, the borderline result was considered as being negative.

For budgetary reasons and because the in-house ELISA is very time-consuming for the laboratory, this test was not applied to all samples and only for the serovars most commonly found in samples from equine eyes. In many cases, this in-house ELISA was initiated only when the MAT result was negative. Serovars for ELISA were selected based on MAT results and the geographic origin of the horse. Between one and four different serovars were used for each sample. The intraocular samples were tested individually with each of the serovars. In the case of a negative MAT result, the ELISA was only performed for the serovars Grippotyphosa and Bratislava. In addition to Grippotyphosa and Bratislava, serovars Canicola, Copenhageni, Icterohaemorrhagiae, and Pomona were also used. For statistical analysis, as with MAT, the respective serovars were not considered for ELISA results. Only the highest absorbance measured for each immunoglobulin class was included in the evaluation. 

A real-time PCR targeting the lipL32 gene of pathogenic *Leptospira* spp. was performed with 1368 intraocular samples.

Not all laboratory tests were performed with every sample. In several cases, only the MAT was ordered initially. In the case of a positive result, this was sometimes left at that for cost reasons, and only in the case of a negative MAT result were the in-house ELISA and PCR performed afterward. In other samples, all tests were performed at once.

### 2.5. Statistical Analysis

Statistical analysis was performed using SPSS for Windows version 28.0. Sensitivity, specificity, and predictive values were calculated as previously described by Richter and Lange [42].

To identify dependencies and statistical relationships among pretest, clinic, and laboratory outcome variables, Spearman’s rank correlation coefficient (ρ) for ordinally scaled characteristics was used. For nominal variables, Pearson’s chi-squared coefficient was used.

The null hypothesis H0 was defined as independent of the two variables tested. An agreement check between the different laboratory tests (MAT, ELISA, PCR, specific immunoglobulins) for positive or negative results was performed using Cohen’s Kappa. Scoring was conducted as suggested by Landis and Koch: κ < 0: “low agreement”; κ 0–0.20: “weak agreement”; κ 0.21–0.40: “sufficient agreement”; κ 0.41–0.60: “moderate agreement”; κ 0.61–0.80: “extensive agreement”; κ 0.81–1.00: “(almost) complete agreement” [43]. 

## 3. Results 

### 3.1. Intraocular Samples

Of the total of 1840 retrospectively examined intraocular specimens, 216 were from healthy eyes without a history of eye disease, 1387 from eyes with ERU, 237 from eyes with other types of uveitis (e.g., phacogenic uveitis, leopard coat pattern uveitis, traumatic uveitis, or chronic iritis) (Table 1). Intraocular samples consisted of 407 aqueous humor samples and 1433 vitreous samples. Right and left eyes were affected with equal frequency. 

### 3.2. Clinical History

**Figure 1:**
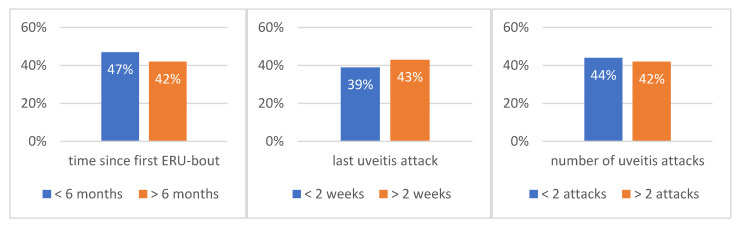
Time period since observation of the first ERU episode (left), time since last ERU episode (middle), and number of uveitic episodes (right) at the time of sampling (*n* = 1387).

According to the medical history, 47% (652/1387) of the eyes affected by ERU suffered from the disease for less than six months and 42% (583/1387) for more than six months. In 39% of the ERU eyes (535/1387), the last episode of uveitis was less than two weeks and in 43% (594/1387) more than two weeks before the time of sampling. In 44% of ERU eyes (604/1387), the number of documented uveitis episodes was two or less, and 48% of ERU-eyes (660/1387) had at least three uveitis attacks and in some cases significantly more than three bouts (Figure 1).

### 3.3. Ophthalmological Findings in ERU 

**Figure 2:**
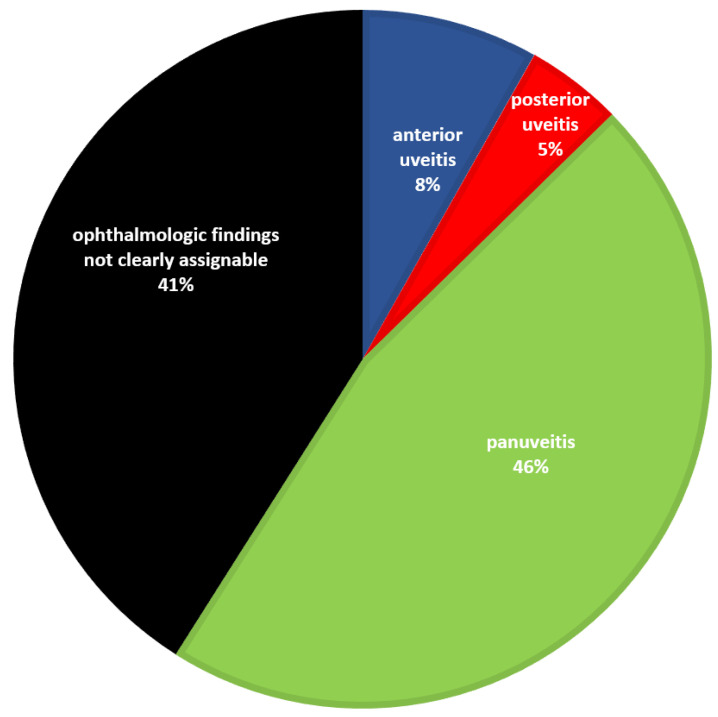
Proportion of ERU eyes with panuveitis, findings that were not clearly assignable, predominantly anterior uveitis, and predominantly posterior uveitis (*n* = 1387). Eyes in which the ophthalmologic findings were not clearly assignable to a uveitis form were in a very early ERU stage and did not yet show clearly attributable ophthalmological findings in the clinically inflammation-free interval. In these cases, either an aqueous humor analysis was performed before vitrectomy or the horses underwent surgery because the preliminary report given by the referring veterinarian was convincing.

Regarding the affected anatomical structures, the ophthalmological findings were classified as anterior uveitis, posterior uveitis, and panuveitis. In 44% (610/1387), no such classification could be made due to the absence of inflammatory signs at the time of examination (Figure 2). 

In addition, the severity of diffuse vitreous haziness and vitreous floaters was assessed (Table 2). In 610 of the ERU eyes, no diffuse vitreous haziness was evident, and in 393 of the ERU eyes, no floaters were evident in the vitreous cavity (Table 2). 

### 3.4. Examination of Aqueous and Vitreous Samples: Detection of Anti-Leptospira Antibodies and PCR Results 

#### 3.4.1. MAT Titers in Intraocular Samples from Eyes Affected by ERU or Another Type of Uveitis, as Well as Healthy Eyes

All intraocular samples (*n* = 1840) were tested by MAT. All 216 intraocular samples from healthy eyes and all 237 intraocular samples from eyes with phacogenic or leopard uveitis were negative in MAT. In ERU eyes, 83% (1064/1281) were MAT-positive. A total of 106 out of 1387 eyes affected with ERU and 1 eye with a leopard coat pattern uveitis had MAT titers of 1:25 or 1:50 and were excluded from further analysis (Table 1). Thus, 1281 intraocular samples from ERU eyes were either MAT-negative or MAT-positive. 

Considering the different titer levels, 42% of the ERU cases (533/1281) had a titer between 1:100 and 1:400, 35% (446/1281) had a titer between 1:800 and 1:3200, and 7% (85/1281) had a titer of 1:6400 or higher. Ophthalmologic examination of eyes affected with ERU and the respective MAT results showed a moderate correlation with vitreous opacities (ρ = 0.382, *p* < 0.001).

**Figure 3:**
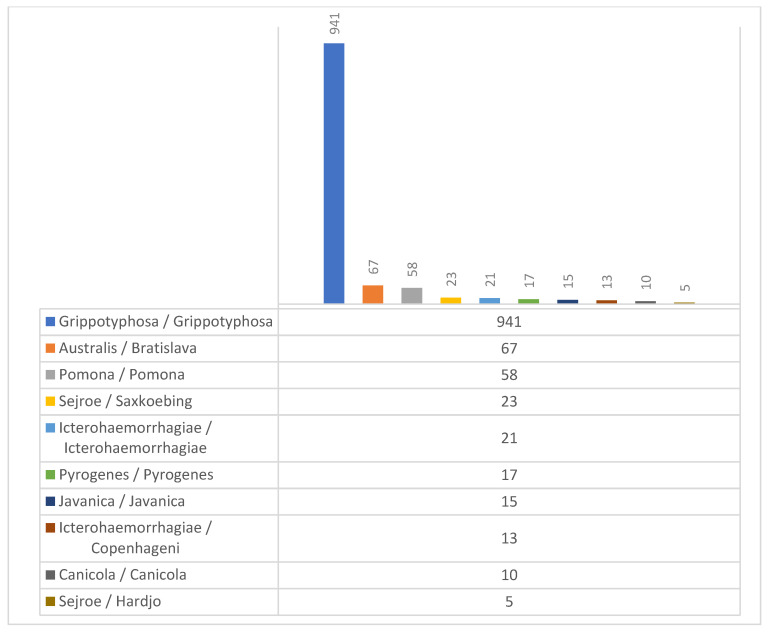
Serovars detected by MAT in the 1170 intraocular specimens from ERU eyes (serogroup/serovar).

Figure 3 depicts the different serovars detected in 1170 intraocular samples from ERU eyes by MAT. The serovars of the low MAT titers (1:25 and 1:50) were included here. The relative frequency of serovars detected by MAT did not change significantly from 2002 until 2017.

#### 3.4.2. Results of the In-House ELISA Using Intraocular Samples from Eyes Affected by ERU or Another Type of Uveitis, as Well as Healthy Eyes

A total of 892 intraocular samples were analyzed by the in-house ELISA for anti-leptospiral antibodies. A total of 113 of these samples were from healthy eyes and 230 samples were taken from eyes with non-leptospiral uveitis. All of these 343 intraocular samples from non-ERU eyes were negative in the ELISA test. However, in 83% (457/549) of samples from ERU eyes, anti-*Leptospira* antibodies could be detected by ELISA. 

Clear ophthalmologic findings related to ERU showed a significant correlation with a positive ELISA result when testing the corresponding intraocular samples (301/338 (89%), *p* < 0.001). When considering individual findings in terms of ERU, panuveitis, the presence of vitreous opacities (regardless of the degree of opacification), and the presence of a high degree of vitreous opacities also correlated significantly with a positive ELISA result in the respective intraocular samples (panuveitis 162/182 (89%), *p* = 0.012; vitreous haze 193/212 (91%), *p* = 0.001; high degree of vitreous floaters 80/87 (92%), *p* = 0.023). However, the degree of the vitreous haze was not associated with the ELISA results.

IgA antibodies correlated strongly with the diagnosis of ERU (κ = 0.749, *p* < 0.001), whereas IgG and IgM antibodies showed moderate (κ = 0.606, *p* < 0.001) and weak correlation (κ = 0.332, *p* < 0.001), respectively, with the diagnosis of ERU.

#### 3.4.3. PCR Results Using Intraocular Samples from Eyes Affected by ERU or Another Eye Disease, as Well as Healthy Eyes 

Of the 1368 intraocular samples tested for leptospiral antigen by PCR, 213 were from healthy eyes and 210 were from eyes with other types of uveitis. All except one of the non-ERU eyes were negative using PCR. The sample from one healthy control eye reacted positive in the PCR. Of the 945 intraocular samples from ERU eyes, 72% (679/945) were positive. 

### 3.5. Comparison of MAT, ELISA, and PCR 

**Figure 4:**
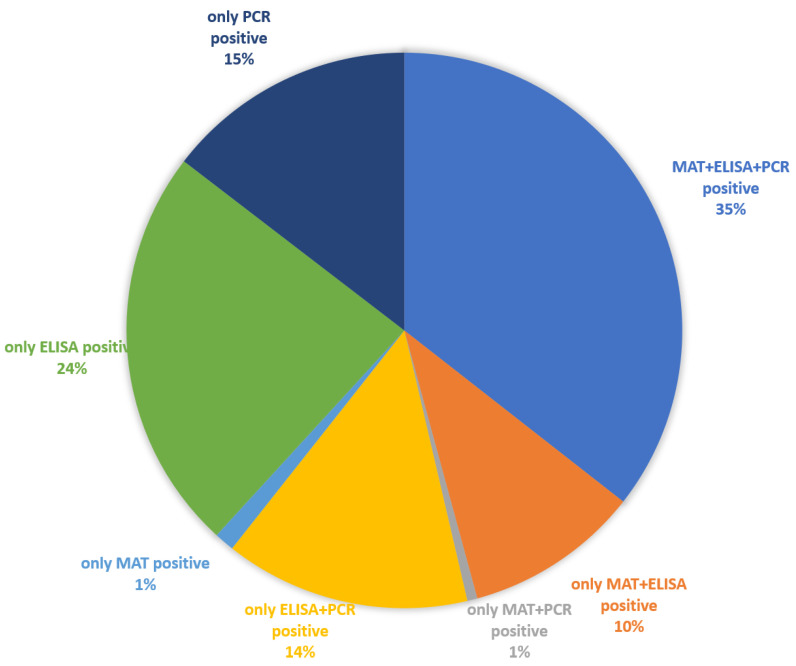
Presence of anti-*Leptospira* antibodies and positive PCR results in intraocular samples from ERU eyes (*n* = 343) (see Table S2).

A total of 343 intraocular specimens from ERU eyes were tested with all three laboratory tests (MAT, in-house ELISA, and PCR) (Figure 4). 

MAT weakly correlated with PCR (κ = 0.241 *p* < 0.001) and ELISA (κ = 0.278 *p* < 0.001). Correlation between ELISA and PCR was very weak (κ = 0.121 *p* < 0.01).

**Figure 5:**
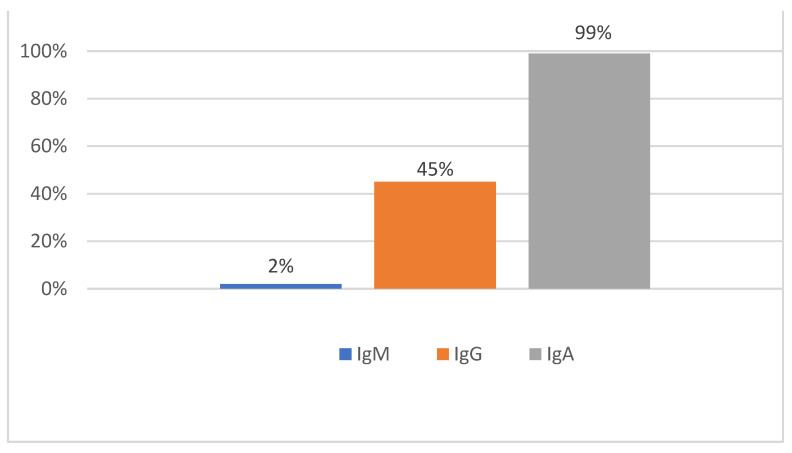
Distribution of immunoglobulin classes using the in-house ELISA for the detection of specific antibodies against the serovars Grippotyphosa and Bratislava (*n* = 457).

If the positive ELISA results are further subdivided into the different antibody classes, antibodies of the IgA class were detectable in almost all samples (99%), followed by significantly fewer positive IgG detection in 45% of the samples and only a small number of samples (2%) with IgM detection (Figure 5). 

In samples from eyes affected by ERU, there was the greatest agreement between the MAT and the in-house ELISA for IgG antibodies (κ = 0.72, *p* < 0.001); for IgA and IgM antibodies, the agreement with MAT was slightly lower (κ = 0.56, *p* < 0.001). 

### 3.6. Sensitivity, Specificity, Positive and Negative Predictive Value

Table 3 shows the sensitivity, specificity, positive, and negative predictive value of MAT, PCR, and ELISA, including the IgA, IgG, and IgM antibodies (numbers see Table S1).

Eyes with another type of uveitis than ERU (e.g., phacogenic uveitis, leopard coat pattern uveitis, traumatic uveitis, chronic iritis) were excluded for diseased and control samples. The inclusion criteria were met by 1497 vitreous samples for the MAT (ERU *n* = 1281, controls *n* = 216), 1159 vitreous samples for PCR (ERU *n* = 945, controls *n* = 214), and 690 vitreous samples for the in-house ELISA (ERU *n* = 549, controls *n* = 113).

## 4. Discussion

The present study convincingly demonstrates the great importance of chronic intraocular leptospiral infection, which is detectable in all stages of ERU as long as the eyes do not yet show significant atrophy or phthisis. All intraocular specimens from ERU eyes in this study were from eyes that were still suitable for vitrectomy, i.e., had not yet shown significant bulbar atrophy or even had not yet shown any ophthalmologic changes at the time of admission during the inflammation-free interval. 

In the earliest studies in the last century using aqueous humor samples from equine eyes affected with recurrent uveitis, very high MAT titers were observed [44,45,46]. PCR did not exist at that time, but successful culturing of leptospires from samples taken from these eyes has been reported in individual cases [45,47,48]. The Goldmann–Witmer coefficient (GWC), which is still cited today, is based on the examination of 26 samples from horse eyes [49]. In the following period, samples from equine eyes suffering from recurrent uveitis were used as a model for the diagnosis of infectious uveitis in humans [50]. At that time, it was recognized that the diagnosis of infectious uveitis cannot be reliably made with serum tests and that the examination of intraocular samples is crucial to making a diagnosis [51,52,53,54,55]. With the development of PCR, important progress had been achieved, which considerably improved the diagnosis of intraocular samples even with small aqueous humor sample volumes [55,56,57,58,59]. 

The GWC is still used for the detection of intraocular antibody production [55,56,58,60,61]. After calculation of the GWC in a large number of equine eyes, intraocular antibody production was detected in almost all cases [62]. At the same time, leptospires were successfully cultured with the intraocular specimens and leptospiral DNA was often detected by PCR—even from intraocular samples which had reacted negatively in MAT [1,62]. When in doubt (very low intraocular antibody titer, no definite ophthalmologic findings, and no conclusive medical history), calculation of the GWC is certainly indicated. On the other hand, after the examination of thousands of intraocular samples from ERU eyes, it has been shown that in horses with typical ERU findings during thorough ophthalmic examination, calculation of the GWC is not always necessary to make a reliable diagnosis. [1].

Some other studies examining intraocular specimens from ERU eyes dealt with end-stage cases of ERU (blind, enucleated, significantly atrophied, or phthitic eyes) [63,64,65,66]. In these end-stages of ERU, the intraocular structures, especially the anterior chamber and vitreous cavity, are no longer in a physiologic configuration. This may be an important reason why the detection of intraocular leptospiral infection was no longer possible in many of these eyes. 

Vitreous specimens obtained during vitrectomies in this study were from ERU eyes in which no preoperative aqueous humor examination had been performed, but in which the indication for vitrectomy had been made on the basis of ophthalmologic findings (either clear preliminary report or clear findings on admission). In these vitreous samples, either anti-*Leptospira* antibodies were detectable or leptospiral DNA was detected by PCR. A few individual cases in which neither antibodies nor a positive PCR result was detected did not meet the inclusion criteria of this study. Whether these samples, which were excluded from the statistics, had reacted false-negative or whether another cause for the uveitis had been present, cannot be determined anymore. The clinical diagnosis of ERU and thus the indication for vitrectomy is therefore not 100% accurate, but still very reliable, if conducted properly, also without prior aqueous humor analysis.

The aqueous humor samples from eyes affected with ERU were exclusively from eyes in which the diagnosis could not be clearly established preoperatively based on the preliminary report or clinical findings alone. If the inflammation has occurred repeatedly, laboratory diagnostic evidence of infection can usually be obtained, but false negative results cannot be ruled out. In these eyes, which are only slightly damaged and not yet damaged, leakage of proteins from the uveal vessels is not to be expected, which is why MAT titers below 1:100 can also be regarded as “positive” [1]. The authors recommend asking the laboratory to also report MAT titers below 1:100 and not to report all values below 1:100 as “negative”. Since these eyes are typically still in very good condition, it is usually not a problem if a uveitis attack occurs again after a “false-negative” aqueous fluid analysis. If this attack is treated carefully, vitrectomy typically still has a good prognosis afterward.

Eyes without ophthalmological changes and, in particular, no posterior synechiae, no diffuse vitreous opacification, and no vitreous floaters were visible at the time of admission were in an early stage of ERU. If these eyes had had anterior uveitis, it had been medicated well, so that no changes in terms of ERU were recognizable in the inflammation-free interval. Over time, the good prognosis of vitrectomy for long-term preservation of vision has led to most horses being taken for surgery at a relatively early stage of disease, which in turn improves the prognosis. On the other hand, in these still apparently undamaged eyes in early ERU stages, it is also not yet possible to assign whether it had rather been anterior uveitis, posterior uveitis, or panuveitis. For this reason, in a relatively high percentage, no anatomical assignment of uveitis was possible (Figure 2) and in many eyes, vitreous opacities were not (yet) present (Table 2).

When comparing the different antibody tests (Section 3.5), it must be taken into account that, for budgetary reasons, not all laboratory diagnostic tests were always performed with each sample and ELISA was often used only when the MAT result was negative. The multiple positive ELISA results indicate the high sensitivity, especially in the detection of immunoglobulin class A antibodies, which were also detectable when MAT was negative. 

On the other hand, the samples used for ELISA were partially preselected (MAT negative) and the comparison of the results of MAT and ELISA is therefore slightly falsified. If the ELISA had always been performed simultaneously with the MAT, it could also be that in individual additional samples only the MAT would have reacted positively and the ELISA would have been negative. However, the high sensitivity and specificity of the detection of immunoglobulin class A antibodies in eyes affected by ERU, which had been described previously [39], could also be confirmed with the larger number of samples available here.

Especially in early stages of ERU, both PCR (depending on where in the vitreous the infection was or exactly where the vitreous sample was taken) and antibody detections can be false negative. Due to the immune privilege of the eye [67,68,69] and the approximately 28 mL large immunological niche in the vitreous cavity [70], immune reactions and thus antibody production can be effectively suppressed at the beginning of the disease. The more the infection spreads inside the vitreous cavity and the more obvious the changes in the eye become, especially the more significant the vitreous haziness is, the more reliable are the laboratory findings for an intraocular leptospiral infection [1,20].

The different results when using different antibody detections with the same intraocular sample could be attributed, for example, to different stages of ERU and individual differences in immune responses to infectious agents. This has earlier been discussed for the commercially available SNAP Lepto-test [38]. 

One reason for the higher sensitivity of MAT compared to PCR could be that the agglutinating antibodies are better distributed in the vitreous fluid and are continuously produced. PCR depends on free-floating leptospiral DNA and might be less sensitive due to leptospiral biofilm production in the vitreous [1,21]. Leptospires are probably not continuously released from the biofilm. However, a better understanding of these mechanisms requires future studies.

A comparison of the results of this retrospective analysis with other studies shows that the figures presented here exceed all previously published numbers considerably. In most other studies, not only were the sample numbers much smaller, but the evidence of intraocular leptospiral infection was also present in lower percentages [32,33,34,35,36], which may be due to various reasons:

- As mentioned above, the samples used in some studies were taken from blinded eyes, often even after enucleation [64,66,71]. When specimens from eyes that have already undergone significant atrophy or phthisis bulbi are examined, there is virtually no vitreous cavity left in which the infection can persist.
- The diluted vitreous samples used in other studies may also have altered the results and led to less reliable laboratory detection of an intraocular leptospiral infection [32,33,35].
- The indication for vitrectomy is less consistently made in some studies and is not limited exclusively to eyes affected by ERU. In these studies, more negatively tested vitreous samples are included in the statistics [35,72].
- The MAT titers considered “positive” vary in different studies and thus influence the results.
- The number of tests performed (and with MAT the number of serovars used) with the intraocular samples has an impact on the results, as in some cases only one of several antibody detections or only the PCR may be positive. The more laboratory diagnostics are invested in, the more reliable the detection of intraocular infection will be.

Thus, how the indication for vitrectomy is made, the technique of sample collection, the condition of the eye at the time of sample collection, the tests performed with the samples, and the cut-off titers all affect the results. All of these points may explain why evidence of intraocular leptospiral infection has led to different results in different studies. 

## 5. Conclusions

The examination of a large number of intraocular specimens from eyes affected with ERU in this study highlights the importance of chronic intraocular leptospiral infection associated with biofilm formation as the cause of this disease. Due to the unique characteristics of the eye (large immunologic niche, immune privilege of the eye) and the ability of leptospires to form biofilm in a very short time [73], there are few initial immune responses and antibody detections, which are not always reliable. Depending on the area in the vitreous from which samples are taken and where leptospiral biofilms are located, PCR may also be false negative. When the etiology of uveitis is not clear, ophthalmological examination, and laboratory testing with an aqueous humor sample should be considered. In order to obtain the most reliable information and to reduce false-negative results, different test procedures should be used. In particular, ELISA studies with detection of specific IgA antibodies can significantly increase sensitivity in the examination of intraocular specimens, provided this ELISA is available.

## Figures and Tables

**Table 1:** Laboratory results of 1840 intraocular samples (1433 vitreous and 407 aqueous fluid samples).

		ERU (*n* = 1387)	Other Types of Uveitis (*n* = 237)	Healthy Eyes (*n* = 216)
**MAT**	tested	1387	237	216
	≥1:100	1064 (83%)	0	0
	1:25 or 1:50 ^1^	106	1	0
	negative	217	236	216
**PCR**	tested	945	210	213
	positive	679 (72%)	0	1 (0.5%)
**ELISA ^2^**	tested	549	230	113
	positive	457 (83%)	0	0

^1^ Intraocular samples with titers of 1:25 and 1:50 in MAT were excluded from the statistical analysis because, although they were below the titer of 1:100 defined as “positive” for this study, they were not “negative” either. More recently, MAT titers below 1:100 are also considered “positive” if there is no presence of aqueous humor and vitreous haziness and thus no relevant “leakage” from the blood into the eye [1]. ^2^ The ELISA was not always performed, but in many cases was only performed as a supplementary test if the MAT was negative. ELISA, especially for immunoglobulin class A antibodies, is often more sensitive than MAT [39].

**Table 2:** Ophthalmological vitreous findings of the 1387 ERU eyes.

Vitreous Findings		Number	%
**vitreous opacit** **y**	none	610	44%
	mild	346	25%
	moderate	265	19%
	severe	166	12%
	total	1387	100%
**vitreous floaters**	none	393	28%
	mild	327	24%
	moderate	343	25%
	severe	324	23%
	total	1387	100%

**Table 3:** Sensitivity, specificity, positive, and negative predictive value for antibody detection and PCR using vitreous samples. All samples were positive in at least one test procedure and had been taken from eyes with typical ophthalmological symptoms in terms of ERU. Thus, no peroperative aqueous analysis has been performed.

Laboratory Test	Sensitivity	Specificity	Positive Predictive Value	Negative Predictive Value
**MAT**	83.1%	100%	100%	49.9%
**PCR**	71.9%	99.5%	99.9%	42.9%
**ELISA**	83.2%	100%	100%	55.1%
**IgA**	82.1%	100%	100%	53.6%
**IgG**	60.3%	100%	100%	34.1%
**IgM**	22.6%	100%	100%	21%

## Data Availability

Not applicable.

## Supplementary Materials

The following supporting information can be downloaded at: https://www.mdpi.com/article/10.3390/vetsci9080448/s1, Table S1: Intraocular samples that met the inclusion criteria for the calculation of sensitivity, specificity, positive and negative predictive value. Table S2: Presence of anti-*Leptospira* antibodies and PCR results in intraocular specimens (*n* = 343) from ERU patients.
