# Supplementary material for: Analysis of 1840 Equine Intraocular Fluid Samples for the Presence of Anti-Leptospira Antibodies and Leptospiral DNA and the Correlation to Ophthalmologic Findings in Terms of Equine Recurrent Uveitis (ERU)—A Retrospective Study
Source: Vet Sci. 2022 Aug 21;9(8):448. doi: 10.3390/vetsci9080448 (PMC9414351; doi:10.3390/vetsci9080448)
Supplement: Supplementary file 1 [file vetsci-09-00448-s001.zip › vetsci-1820563-supplementary.pdf]

Table S1: Intraocular samples that met the inclusion criteria for the calculation of sensitivity, specificity, positive and negative predictive value.

Samples from	MAT		ELISA IgA		ELISA IgG		ELISA IgM		ELISA all tests		PCR	
	≥ 1:100	neg	pos	neg	pos	neg	pos	neg	pos	neg	pos	neg
ERU eyes	1,064	217	451	98	331	218	124	425	457	92	679	266
healthy control eyes	0	216	0	113	0	113	0	113	0	113	1	213

Table S2: Presence of anti-Leptospira antibodies and PCR results in intraocular specimens (n= 343) from ERU patients. (pos. = positive; neg. = negative)

results of laboratory tests	number positive / number tested	percentage positive
MAT pos. + ELISA pos. + PCR pos.	122/343	35
MAT pos. + ELISA pos. + PCR neg.	35/343	10
MAT pos. + PCR pos. + ELISA neg.	2/343	1
ELISA pos. + PCR pos. + MAT neg.	49/343	14
only MAT pos.	4/343	1
only ELISA pos.	81/343	24
only PCR pos.	50/343	15
